# Supplementary material for: Comparison of Chayote (Sechium edule (Jacq.) Sw.) Accessions from Mexico, Japan, and Myanmar Using Reproductive Characters and Microsatellite Markers
Source: Plants (Basel). 2023 Jan 19;12(3):476. doi: 10.3390/plants12030476 (PMC9919900; doi:10.3390/plants12030476)
Supplement: Supplementary file 1 [file plants-12-00476-s001.zip › Supplementary Figure.pdf]

Supplementary Materials

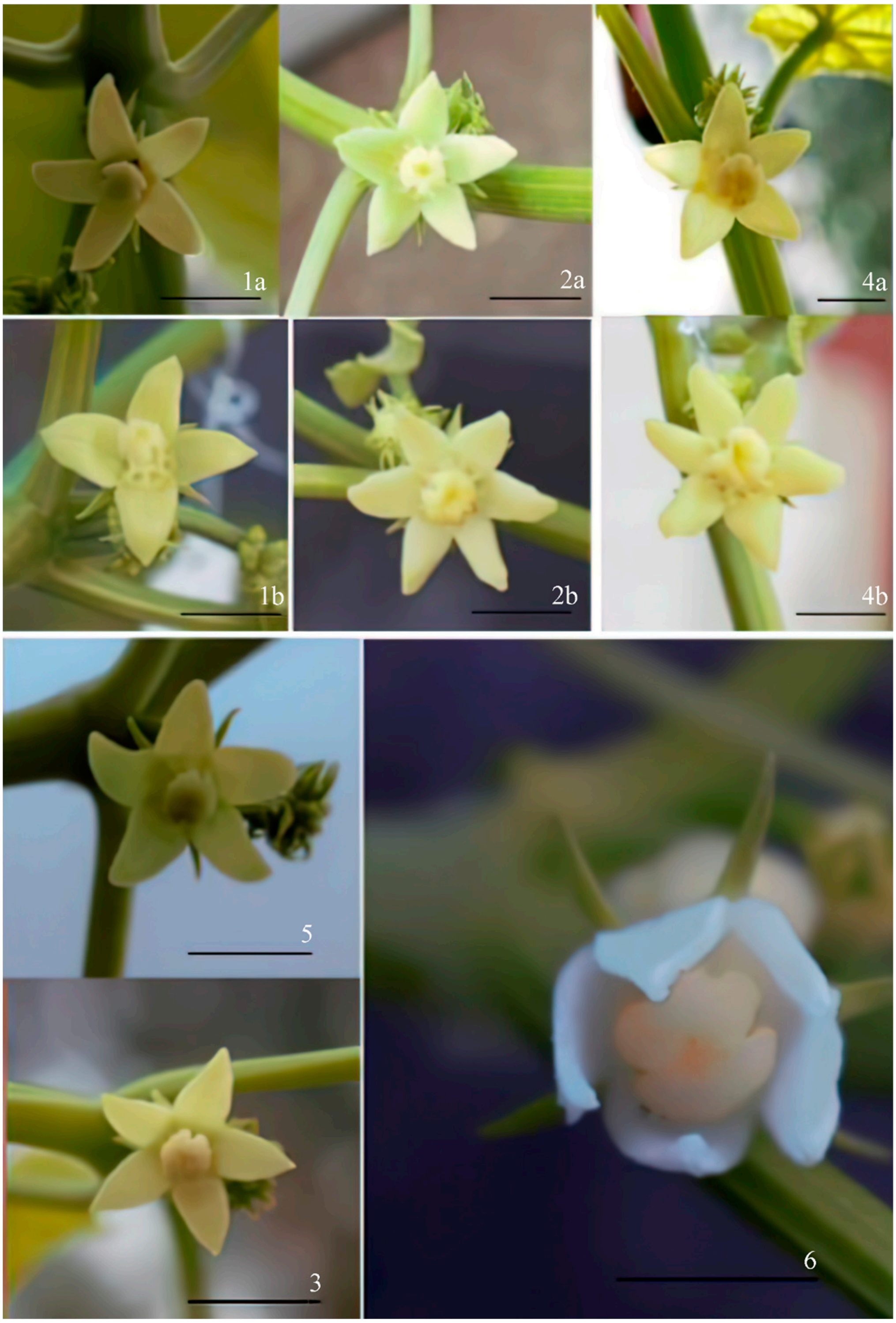

Figure S1. Shape variation observed in female flower of Japanese and Mexican *S. edule* varieties. 1-5, Mexican *S. edule* varieties (**1a,b**, *virens levis*; **2a,b**, *nigrum xalapensis*; **3**, *nigrum minor*; **4a,b**, *albus levis*; **5**, *albus dulcis*); **6**, Japanese *S. edule* variety *albus levis*. The bars represent 0.5 cm.

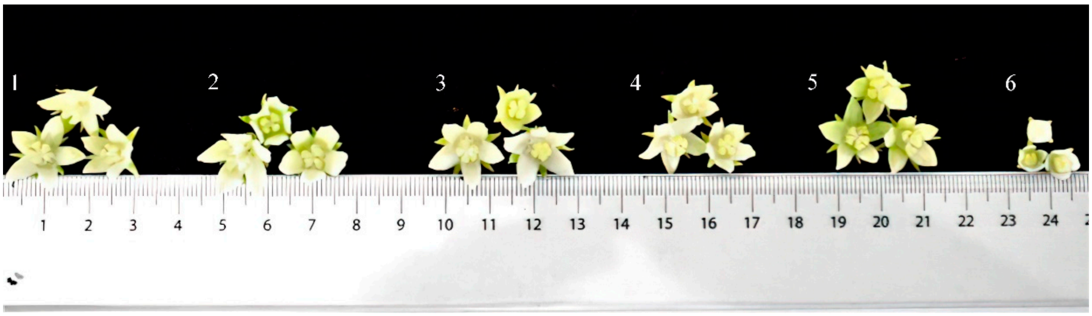

Figure S2. Shape variation observed in male flower of Japanese and Mexican *S. edule* varieties. 1-5, Mexican *S. edule* varieties (**1**, *virens levis*; **2**, *nigrum xalapensis*; **3**, *nigrum minor*; **4**, *albus levis*; **5**, *albus dulcis*); **6**, Japanese *S edule* variety *albus levis*.

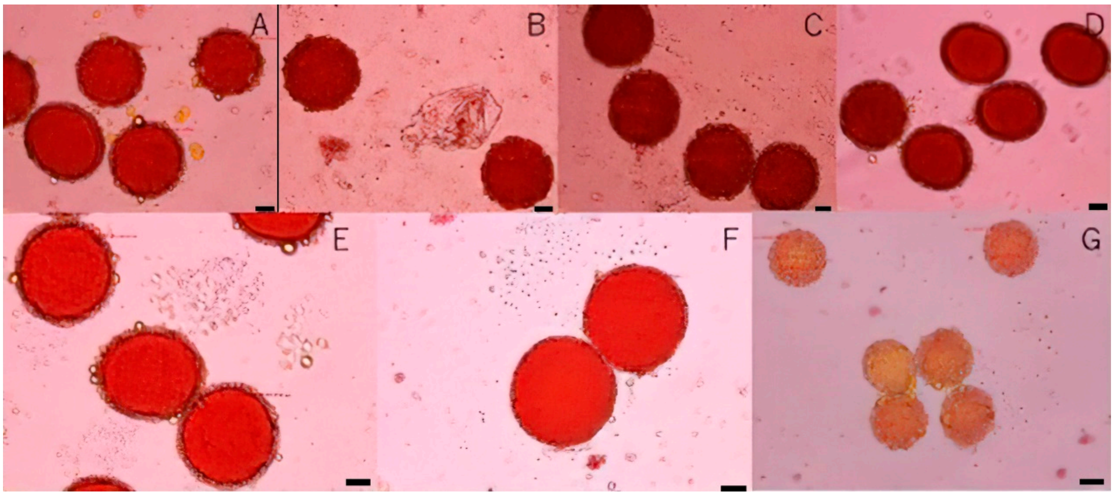

Figure S3. Pollen photos of 5 Mexican and 1 Japanese *S. edule* varieties. **A**, **B**, **C**, **D**, and **E** represent pollen of 5 Mexican *S. edule* varieties *virens levis*, *nigrum xalapensis*, *nigrum minor*, *albus dulcis*, and *albus levis*; **F**,**G** represents Japanese *S. edule* varieties *albus levis* and virens levis. The black scale bars indicate 20μm.

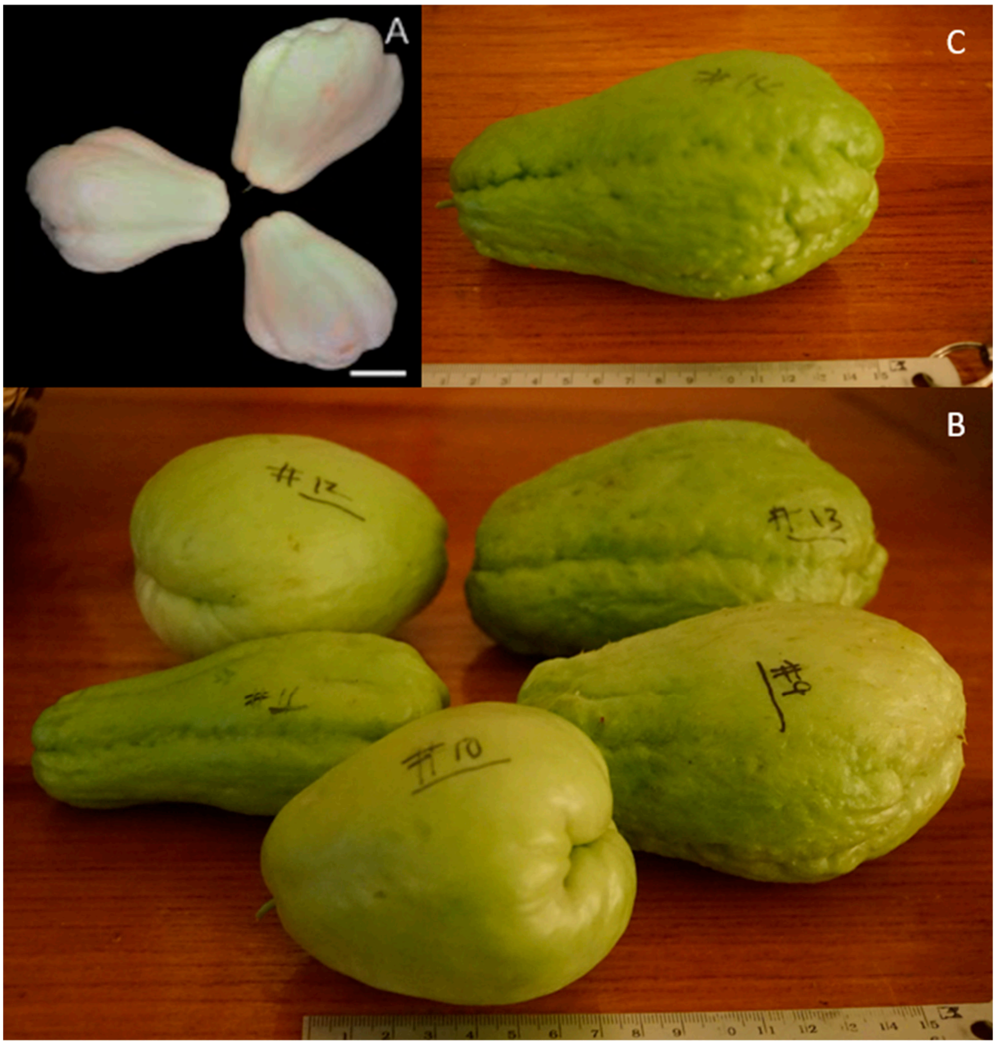

Figure S4. Mature fruit of representative Japanese and Myanmar accessions. **A**: KW019, Japanese *albus levis* accession, the bar represents 2 cm. **B**,**C:** #10=KNW10, #12=KNW12, Myanmar *albus levis* accessions; #9=KNW09, #11=KNW11, #13=KNW13, #14=KNW14, Myanmar *virens levis* accessions.

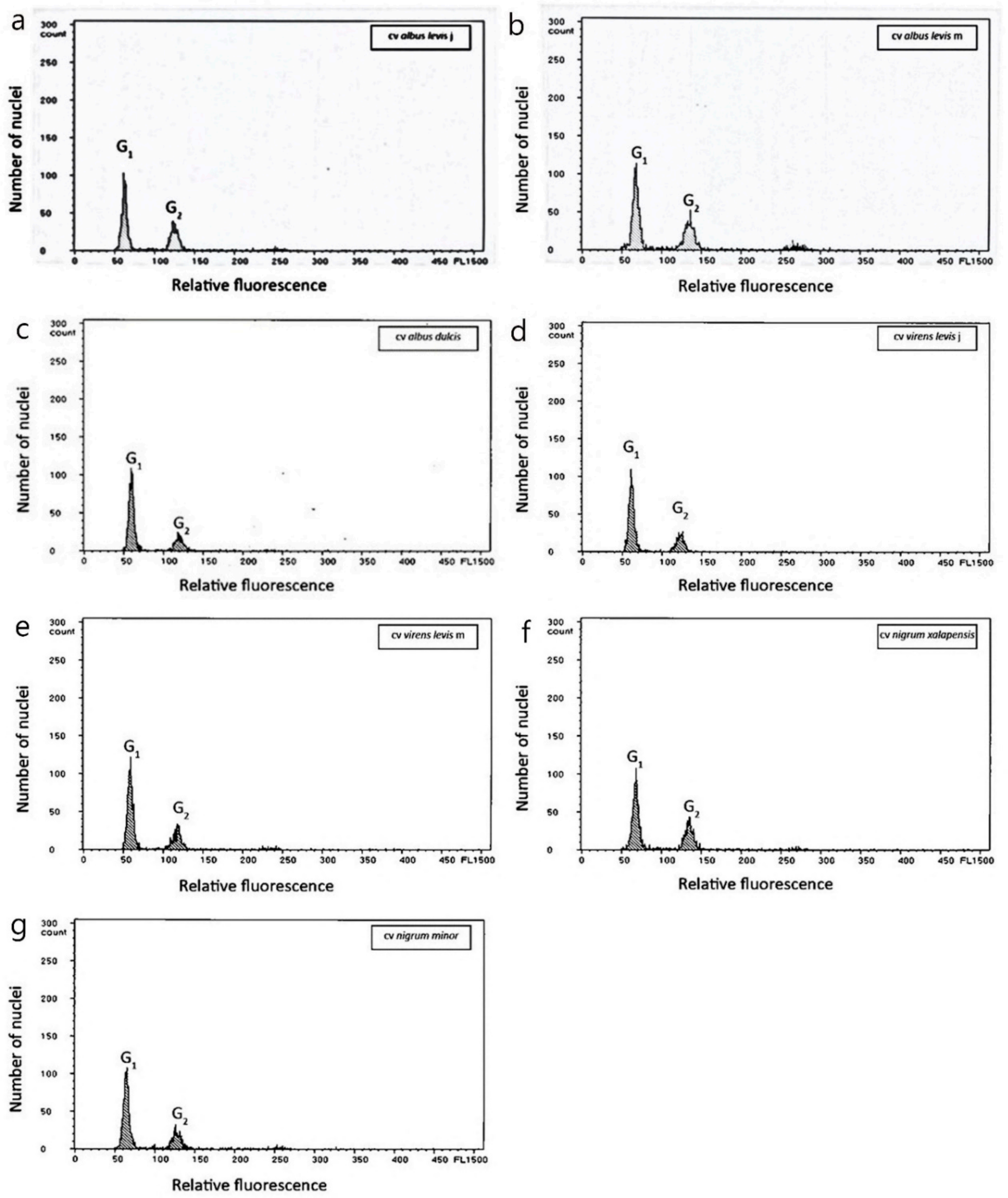

Figure S5. Relative fluorescence intensity histogram in Japanese (j) and Mexican (m) *Sechium edule* varietal group accessions. (a) KW019, *albus levis* j (b) KW017, *albus levis* m (c) KW018, *albus dulcis* m (d) KW020, *virens levis* j (e) KW014, *virens levis* m (f) KW015, *nigrum xalapensis* m (g) KW016, *nigrum minor* m

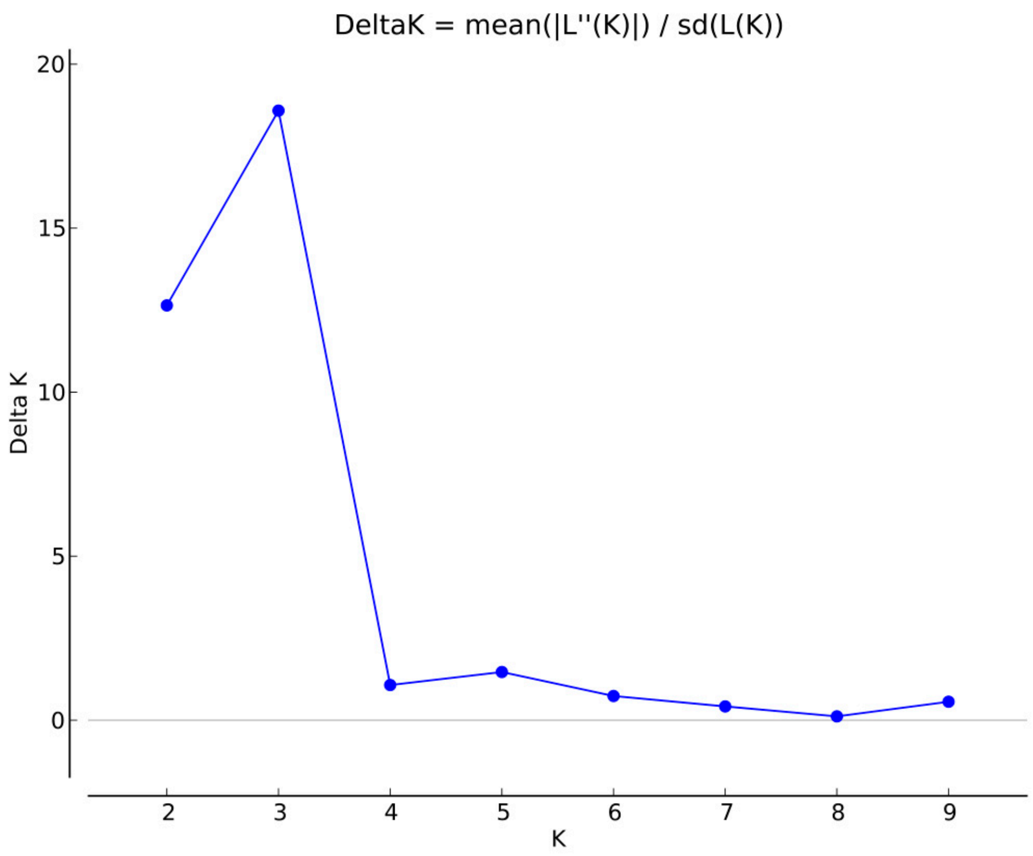

Figure S6. Delta K calculated by Structure Harvester using Evanno's method, showing peak value at K = 3.

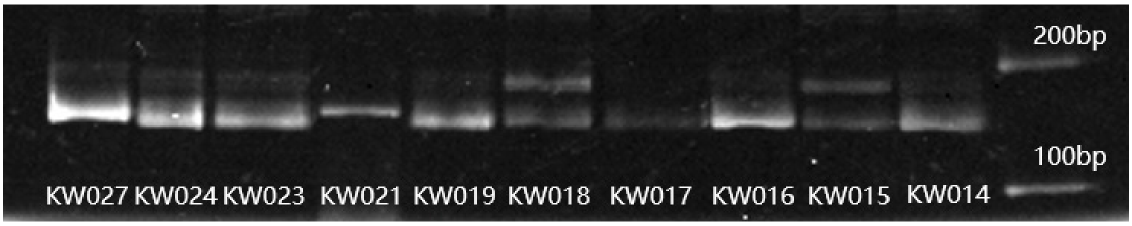

Figure S7. An example of PAGE genotyping results (Sed03 with expected size of 144bp). The rightmost lane is ladder showing 100bp and 200bp.
